# APH-2 and Tax expression are correlated with a HTLV-2 proviral load but not with lymphocytosis

**DOI:** 10.1186/1742-4690-8-S1-A184

**Published:** 2011-06-06

**Authors:** Estelle Douceron, Zhanna Kaidarova, Paola Miyazato, Masao Matsuoka, Edward L Murphy, Renaud Mahieux

**Affiliations:** 1 Equipe Oncogenèse Rétrovirale, INSERM-U758 Virologie Humaine, Lyon, cedex 07, 69364, France; 2 Ecole Normale Supérieure de Lyon, Lyon, cedex 07, 69364, France; 3 IFR 128 Biosciences Lyon-Gerland, Lyon, cedex, 0769364, France; 4 University of California San Francisco, Blood Systems Research Institute, San Francisco, California, USA; 5 Laboratory of Virus Immunology, Institute for Virus Research, Kyoto University, Kyoto, 606-8507, Japan

## 

The recent discovery of HBZ, an antisense protein, encoded by HTLV-1 allowed a new way of understanding how HTLV-1 induces the development of adult T cell leukemia/lymphoma (ATLL). HBZ mRNA is expressed in all HTLV-1 patients tested regardless of their clinical status. Furthermore HBZ mRNA level is positively correlated to the HTLV-1 proviral load and it involved in infected T cell proliferation. The HTLV-2 homolog of HBZ, APH-2, also represses the viral transcription from the 5' LTR. We therefore quantified APH-2 and Tax mRNA levels as well as proviral load in a series of 51 blood samples obtained from the HTLV Outcomes Study (HOST) cohort. These samples were divided in low, intermediate and high proviral load (PVL) groups. We first show that APH-2 was expressed in most (94%) samples, while Tax was expressed mostly in the high PVL group. A positive correlation was observed between PVL and Tax and between PVL and APH-2. Although lymphocytosis is commonly observed among HTLV-2 carriers, we also demonstrate that APH2, contrary to HBZ does not promote cell proliferation in vitro. These results were confirmed in vivo since we did not observe a correlation between APH-2 level and the lymphocyte count. Our results therefore demonstrate that APH-2 is frequently expressed in vivo in HTLV-2 carriers. However, and contrary to HBZ, APH-2 does not promote cell proliferation.

